# Experiences and knowledge of nurses, occupational therapists, pharmacists and physiotherapists about certifying fit notes: a UK-wide survey

**DOI:** 10.1136/bmjopen-2024-092211

**Published:** 2025-05-15

**Authors:** Jade Kettlewell, Diane Trusson, Katie Powers, Avril Drummond, Claire Anderson, Gill Phillips, Jain Holmes, Kate Radford, Nick Pahl, Shan Martin, Stephen Timmons, Denise Kendrick

**Affiliations:** 1 Centre for Academic Primary Care, University of Nottingham School of Medicine, Nottingham, UK; 2 Mental Health and Clinical Neuroscience, University of Nottingham School of Medicine, Nottingham, UK; 3 NIHR Nottingham Biomedical Research Centre, Nottingham, UK; 4 University of Nottingham School of Medicine, Nottingham, UK; 5 University of Nottingham School of Health Sciences, Nottingham, UK; 6 Division of Pharmacy Practice and Policy, University of Nottingham School of Pharmacy, Nottingham, UK; 7 PPI Representative, University of Nottingham School of Medicine, Nottingham, UK; 8 Centre for Rehabilitation and Ageing Research, University of Nottingham School of Medicine, Nottingham, UK; 9 Society of Occupational Medicine, London, UK; 10 Nottingham University Business School, Nottingham, UK

**Keywords:** Nurses, Pharmacists, Surveys and Questionnaires, Primary Care, Occupational Health Services, MEDICAL EDUCATION & TRAINING

## Abstract

**Abstract:**

**Objective:**

To identify facilitators and barriers to fit note certification among nurses, occupational therapists, pharmacists and physiotherapists (NOPPs), and identify ongoing training needs.

**Design:**

An online survey informed by the Theoretical Domains Framework (TDF) was used to gather data from NOPPs to identify implementation barriers and personal, social and environmental influences on fit note certification.

Data were analysed using descriptive statistics. Mean TDF domain scores were calculated (mean scores ≤3.5 indicated barriers, ≥5 indicated facilitators). Free-text data were thematically analysed using the TDF.

**Setting:**

United Kingdom.

**Participants:**

The survey was completed by 198 respondents: physiotherapists (n=66, 33%), occupational therapists (n=49, 25%), nurses (n=44, 22%), pharmacists (n=39, 20%).

**Results:**

Only 47 (24%) of survey respondents had certified fit notes; 66 (37%) had completed training, most pharmacists had done neither. TDF analysis indicated three barriers: 1) ‘skills’ (being able to certify, review and practice completing fit notes) (mean=3.32, SD=0.75, 95% CI 1.84, 4.80); 2) ‘goals’ (the level of priority given to fit note completion) (mean=3.22, SD=0.51, 95% CI 2.21, 4.22); 3) ‘memory, attention and decision processes’ (disagreeing with the statement: ‘certifying fit notes is something I do automatically’) (mean=2.73, SD=0). Free-text comments suggested that low ‘skills’ rates may be due to lack of opportunity to do training. The low priority afforded to completing fit notes, which was not done automatically as part of their role, may reflect the lack of organisational policies/guidelines or priorities.

The only facilitator identified was ‘belief about consequences’ (mean=5.74, SD=0.12, 95% CI: 5.50, 5.98). Participants believed that certifying fit notes was useful and worthwhile.

**Conclusions:**

Legislation allowing NOPPs to undertake fit note certification does not appear to have been successfully implemented. Further resources are required to provide NOPPs with the necessary skills/confidence (e.g., via training) to certify fit notes, supporting more patients to return to and remain in work.

**STRENGTHS AND LIMITATIONS OF THIS STUDY**

- We were able to recruit from all four professions able to certify fit notes.
- Online survey facilitated access to potential participants in NHS and private practice but made response rate calculations impossible.
- The response rate was low, particularly from nurses and pharmacists, who appeared most difficult to access.
- The Theoretical Domains Framework identifies personal, social and environmental influences that would be useful to explore in semistructured interviews with the same professional groups.

## Background

 Fitness to work certification is a common activity in primary care in the UK, with general practitioners (GPs) issuing over 11 million fit notes between 2022 and 2023.^1^ The fit note replaced the ‘sick note’ in 2010, with an aim of reducing avoidable sickness absence and facilitating return-to-work (RTW) for people experiencing health problems.^1 2^ In contrast to the previous ’sick note’, which simply stated that a patient was unable to work and how long she/he should be absent, the fit note requires GPs to discern whether an individual is ‘not fit’ or ‘may be fit’ to RTW.^3^ If ‘may be fit’ is selected, the fit note should also include at least one suggested reasonable adjustment (or ‘work solution’) that might facilitate the employee’s RTW (e.g, phased return, reduced hours and/or amended duties).^4 5^ Employees must obtain a fit note to certify an indefinite period of sick leave following the first 7 days of self-certified absence from work.

Participation in work has been shown to be beneficial to people’s mental and physical health^6^ and also benefits the economy. It is, therefore, important to support patients to RTW as soon as it is safe and appropriate, even if they cannot work at full capacity.^7 8^ This has become a pressing issue in the UK,^9^ with increasing numbers of people who are economically inactive due to long-term sick absence.^10^ Furthermore, the longer someone is signed off work, the less likely they are to RTW.^6 7^

The reasonable adjustments in a completed fit note aim to expedite RTW. However, recommending work adjustments requires an understanding of the person’s functional capability to carry out the responsibilities required by their job role, as well as the impact that their health condition might have on these responsibilities.^11^ Previous research suggests that GPs feel underskilled in this area, and they have identified a need for ongoing and mandatory training.^12 13^ However, although GPs are generally aware of learning resources to aid their knowledge and ability to certify fit notes, competing demands on their time and low priority afforded to sickness certification means that use of these learning resources is limited.^13^ In addition, the length of GP consultations and increasing use of telephone consultations may make RTW assessments difficult.^7 11 14^

GP surgeries in the UK face significant pressure due to the decline in full-time GPs, combined with an increase in patients requiring care.^15 16^ Fit note completion is a frequent and time-consuming task for GPs. Consequently, new legislation in 2022 allowing nurses, occupational therapists (OTs), pharmacists and physiotherapists (NOPPs) to certify fit notes was proposed to alleviate pressure on GPs.^17 18^ There are also clear benefits for patient care when NOPPs can use their skills to provide tailored RTW advice and make better use of the ‘may be fit to work’ section of a fit note.^7 14 18^ For example, Drummond *et al*’s feasibility study of occupational therapy-led vocational therapy in two primary care centres found that there was a positive reduction in sickness absence in patients.^19^ The authors concluded that OTs were well placed to deal with health-related work issues and that they should be able to complete fit notes.

A study of challenges and learning and development needs relating to fit note completion gathered data from 21 first contact practitioner physiotherapists involved in the management of musculoskeletal conditions in primary care.^20^ Using the nominal group technique method, they identified challenges relating to having sufficient time to complete fit notes and providing evidence-based work advice. However, they also felt that with training (particularly on the legal aspects of fit notes), first contact practitioner physiotherapists may be ideally suited to provide supportive conversations about work.^20^ Although there are currently no specific studies around fit note completion by nurses, OTs and pharmacists, there have been studies of task reallocation within the medical workforce. For example, extending nursing roles to include prescribing, a role that was traditionally the domain of medical professionals.^21 22^ In their systematic review, Niezen and Mathijssen^21^ identified four analytical themes of facilitators or barriers to task reallocation: (1) knowledge and capabilities, (2) professional boundaries, (3) organisational environment and (4) institutional environment. In the context of primary care, Wang *et al* identified similar barriers and enablers to implementing clinical practice guidelines at individual healthcare provider and organisational levels.^23^

Although there have been studies of GPs’ experiences of completing fit notes,^3 12^ and challenges and educational needs of first contact practitioner physiotherapists,^20^ there has been no research to date which includes all four professional groups newly able to certify fit notes. Therefore, the impact that legislation enabling NOPPs to certify fit notes will have on their roles and patients’ ability to RTW remains unclear. There is a timely need to explore the experiences and perspectives of NOPPs in certifying fit notes and to understand barriers and facilitators to their completion. Consequently, this study aimed to identify behavioural determinants (i.e., barriers and facilitators) that influence NOPPs’ certification of fit notes using an online survey.

## Methods

An online survey was used to identify the experiences and perspectives of NOPPs to determine experience, training and barriers/facilitators to fit note certification. The online survey was developed using Qualtrics (Provo, Utah, USA).

### Survey design

The survey was developed using the Theoretical Domains Framework (TDF).^24^ The TDF provides a theoretical lens for identifying personal, social and environmental influences on healthcare professionals’ behaviour.^24^ The TDF has been used previously to identify and categorise influencing factors when implementing clinical practice guidelines into primary care.^23^ The TDF incorporates 33 theories of behaviour change into 14 domains, namely: knowledge; skills; social/professional role and identity; beliefs about capabilities; optimism; beliefs about consequences; reinforcement; intentions; goals; memory, attention and decision processes; emotion; environmental context and resources; social influences and behavioural regulation.^24^ The survey comprised 52 statements linked to each of these 14 domains. Each statement was scored on a 7-point Likert scale, where 7=strongly agree and 1=strongly disagree.^25^ Free-text boxes were available at the end of the survey for participants to provide additional information about barriers/facilitators, suggestions for improving training and any other comments. The survey was circulated to the research team, including two patient and public involvement (PPI) members, to get agreement on the content before the link was distributed more widely (see online supplemental file 1 for survey).

### Survey sample

The sample comprised the four professional groups eligible to certify fit notes: NOPPs. A sample size calculation indicated that a sample of 19 per professional group (i.e., 76) is required to estimate an overall TDF domain mean with 95% CI and a precision of 0.5, based on an SD of 1.^26^ The SD is based on a previous study using a TDF survey to identify behavioural factors affecting implementation.^27^ TDF mean domain scores can range from 1 to 7. With 19 participants per group and an SD of 1, the study would have 80% power at the 5% significance level to detect a 1-point difference in mean domain scores between two professional groups.^26^

An electronic link to the survey hosted on Qualtrics (Provo, Utah, USA) was circulated to relevant professional bodies (e.g., Royal College of Occupational Therapists (RCOT), Royal Pharmaceutical Society, Chartered Society of Physiotherapists, Royal College of Nursing, Society for Occupational Medicine) and specialist groups (e.g., RCOT Special Section—Work) across the UK through the study team’s existing contacts, and through academic and professional networks. Social media (e.g., via X/Twitter) facilitated access to a diverse group of healthcare professionals, including those who were self-employed or with no existing links with the research team.

Participants were included in the study if they had current registration and were employed or self-employed as a nurse, OT, pharmacist or physiotherapist. Although the original survey stipulated that participants needed to have fit note experience, this requirement was later dropped (after amending the protocol) to encourage participation in the study. Subsequently, the wording on the survey was altered to state that participants could complete the survey whether they had experience of certifying fit notes or not.

On accessing the survey, participants were presented with the study information sheet. Consent was indicated by ticking an ‘I consent to take part’ box. Participants were informed that the data collected would be treated confidentially and stored securely. Participants could choose to withdraw from the study at any point, but any data that had already been collected could not be erased and would be used in the final analysis. Survey respondents were given the option to provide their email address to enter a prize draw but were assured that their survey responses would remain anonymous. Participants also provided contact information if they were willing to be interviewed in a subsequent part of the study.

### Data analysis

Survey data were analysed using descriptive statistics. Mean TDF question and domain scores, SD and 95% CIs were calculated and summarised. Mean domain scores of ≤3.5 indicated ‘substantial barriers’ to fit note certification and scores of ≥5 indicated ‘enabling facilitators.’ Data were collated and analysed as an overall group with exploratory analyses conducted to compare domain scores between occupational groups using unpaired t-tests or their non-parametric equivalent, depending on assumptions being met. Comparisons were made between participants employed at different sites (e.g., hospitals, GP practices). The overall response rate to TDF-specific questions was lower than expected (32%–57%). To explore possible explanations for this, we also compared two groups: group A (respondents who completed at least one TDF-specific question) and group B (those who did not complete any TDF-specific questions).

Missing data have been clearly indicated in the relevant tables. Percentages of responses were calculated based on the number of answers provided for each question, rather than the total population of survey respondents, to ensure that results accurately reflect the available data. For specific questions where responses were missing, individuals who did not answer were excluded from the analysis of that particular question, ensuring that missing data did not distort findings. Means, SDs and 95% CI have been calculated based on the available responses and do not include missing data.

Qualitative data from free-text comments were exported from the survey and thematically analysed using the framework approach.^28^ The analysis framework was informed by TDF to explore barriers and facilitators to fit note certification in further detail. Authors DT and JK independently coded the data and mapped findings to the TDF domains, then discussed for agreement on key themes. Both quantitative and qualitative data were presented to the wider study group (including two PPI authors), and the interpretation of findings was discussed until a consensus was reached.

### Patient and public involvement

The research team included two PPI representatives (authors GP and SM) who were involved throughout the study. They contributed to the development of the study proposal and applying for funding. They were also involved in developing and piloting the survey, participant recruitment, discussion of the findings (see above) and reviewing the study outputs.

## Results

### Participants

The survey was completed by 198 participants. There were sufficient participants from the targeted professions to meet the required sample size (i.e., at least 19 per professional group). Most participants were physiotherapists (n=65, 33%), followed by OTs (n=49, 25%), nurses (n=44, 22%) and pharmacists (n=39, 20%).

Most participants were employed by the National Health Service (NHS) (n=166, 84%), with others working in private practice (n=34, 17%) or self-employed (n=24, 12%). No option was selected by three participants. Several participants provided more than one response (e.g., working in the NHS and privately); consequently, the total exceeded the number of survey participants. Similarly, there were often multiple answers to the question regarding which setting(s) participants worked in. Most worked in a hospital (n=87, 44%), GP surgery (n=53, 27%) or in a community team (n=47, 24%). A variety of other healthcare settings were also represented, including health centres, private clinics/hospitals and urgent care centres.

The majority of participants (n=102, 52%) had been qualified for over 15 years; 34 (17%) were qualified for 11–15 years, 43 (22%) were qualified for 6–10 years and 20 (10%) were between 0 and 5 years postqualification. Although there was a wide range of expertise among the survey participants, most indicated that they had no specialist clinical area, but treated patients with general health conditions (n=47, 24%). The main areas of expertise were musculoskeletal (n=41, 21%), neurological conditions (n=31, 16%) and mental health (n=16, 8%). For details of the survey participants, see table 1.

**Table 1:** Demographic details of the survey participants

	Number of participants (%) n=198*
Healthcare/service provider role	
Physiotherapist	65 (33)
Pharmacist	39 (20)
Occupational therapist	49 (25)
Nurse	44 (22)
No profession selected	1 (1)
National Health Service (NHS), private or self-employed?†	
NHS	166 (84)
Private practice	34 (17)
Self-employed	24 (12)
No option selected	3 (2)
Location of work/where treat patients†	
General practice	53 (27)
Hospital	87 (44)
Community team	47 (24)
Health centre	6 (3)
Patient’s home	12 (6)
Community pharmacy	11 (6)
Private clinic/practice	7 (4)
Outpatient clinic	4 (2)
Academia/research	3 (2)
Integrated care board	2 (1)
Occupational health	2 (1)
Community hospital	1 (1)
Private hospital	1 (1)
Urgent care centre	1 (1)
Other‡	6 (3)
Years qualified	
0–5 years	20 (10)
6–10 years	43 (22)
11–15 years	34 (17)
Over 15 years	102 (52)
Main area of expertise	
No specialist area—general	47 (24)
Musculoskeletal	41 (21)
Neurological conditions	31 (16)
Mental health	16 (8)
Geriatrics/falls prevention/frailty	10 (5)
Cardiovascular	8 (4)
Pain	6 (3)
Respiratory	5 (3)
Injuries	4 (2)
Major trauma	4 (2)
Paediatrics	4 (2)
Critical care	4 (2)
Cancer	3 (2)
Orthopaedic	2 (1)
Emergency medicine	2 (1)
Women’s health	2 (1)
Substance and alcohol misuse/addictions	2 (1)
Acute medicine	1 (1)
Occupational health	1 (1)
Research	1 (1)
Other§	4 (2)

* Percentages rounded to nearest whole number, hence does not sum to 100%.

† Participants were able to provide more than one response to this question, therefore, total equals more than 198 and greater than 100%.

‡ ‘Other’ locations included charity, early supported discharge team, mediocolegal, social care, county council and occupational health.

§ ‘Other’ areas of expertise included cystic fibrosis, HIV and hepatitis, pelvic health, long covid.

### Fit note certification experience and training

Quantitative data showed that just under a quarter (n=47, 24%) of survey participants had experience of certifying fit notes. Of these, most were physiotherapists (n=19, 29%). There were equal numbers of OTs and nurses with fit note certification experience (n=12 per group), which equated to 24% of OTs and 27% of nurses. Only 4 (10%) of the 39 pharmacists who completed the survey had experience of certifying fit notes.

One-third of respondents, 37% (n=66), had attended fit note training. Training was more common among OTs (n=22, 45%) than physiotherapists (n=24, 36%), nurses (n=14, 32%) and pharmacists (n=6, 15%). Further details are shown in table 2.

**Table 2:** Fit note certification and training

Experience of certifying fit notes	Number of participants (% total)
No	151 (76)
Yes	47 (24)

Experience of certifying fit notes based on profession	Number of participants (% per profession)	
	No experience	Has experience
Nurse (n=44)	32 (73)	12 (27)
Occupational therapist (n=49)	37 (76)	12 (24)
Pharmacist (n=39)	35 (90)	4 (10)
Physiotherapist (n=66)	47 (71)	19 (29)

Attended fit note training based on profession	Number of participants (% per profession)		
	Not attended training	Attended training	No option selected
Nurse (n=44)	26 (59)	14 (32)	4 (9)
Occupational therapist (n=49)	24 (49)	22 (45)	3 (6)
Pharmacist (n=39)	30 (77)	6 (15)	3 (8)
Physiotherapist (n=66)	37 (56)	24 (36)	5 (8)

Experience of fit note completion based on main location of work	Number of participants(% total with fit note experience n=47)
General practice	25 (53)
Hospital	12 (26)
Community	5 (11)
Other (e.g., private practice, occupational health)	5 (11)

Participants working in general practice (n=53) were most likely to have attended fit note training (n=29, 55%) and to have experience of completing fit notes (n=25, 47% of those with experience). Of these, most (n=11, 44%) were physiotherapists; the rest were nurses (n=8, 32%), OTs (n=4, 16%) and pharmacists (n=2, 8%). 14 participants working in a hospital setting had attended fit note training; 12 had experience of completing fit notes. The 14 who were working in hospital who had training included OTs (n=5, 35%), nurses (n=4, 28%), physiotherapists (n=3, 21%) and pharmacists (n=2, 14%).

Four participants with fit note experience worked in the community, including two physiotherapists, one nurse and one OT. Nine participants worked in other locations (including private practice and occupational health). These were physiotherapists (n=7, 78%) and OTs (n=2, 22%). Several participants indicated that they worked in more than one location.

### TDF-specific questions

**Figure 1:**
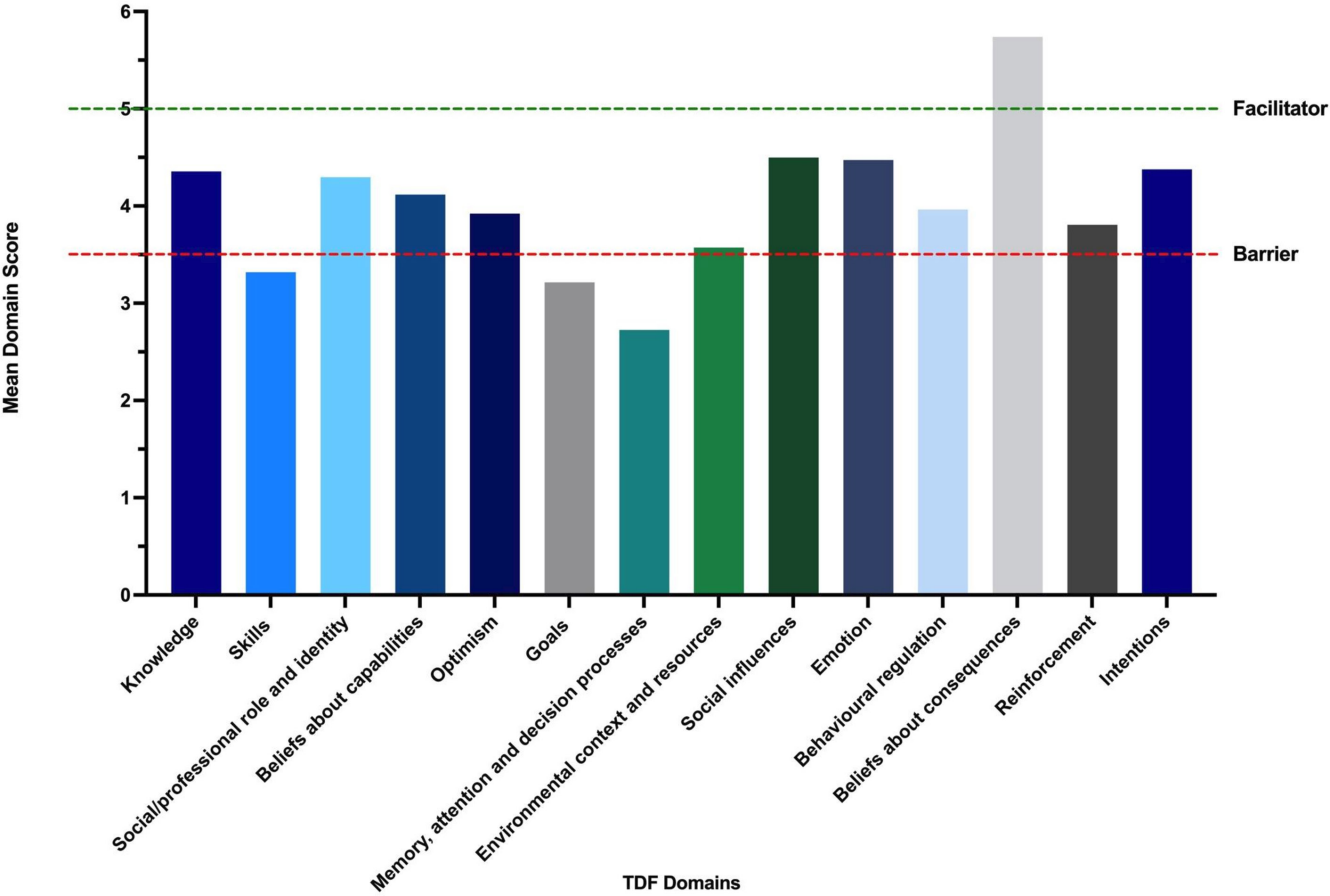
Mean TDF domain scores indicating barriers and facilitators to fit note certification. Barriers and facilitators are indicated by horizontal dotted lines. A ‘substantial barrier’ is defined as a mean domain score of ≤3.5 and facilitator defined as a mean domain score of ≥5*.* TDF, Theoretical Domains Framework*.*

The proportion of responders completing TDF-specific questions ranged from 32% to 57%. According to these responses, the main barriers to certifying fit notes, based on mean domain scores of 3.5 or less, were ‘skills’ (mean=3.32, SD=0.75, 95% CI 1.84, 4.80), ‘goals’ (mean=3.22, SD=0.51, 95% CI 2.21, 4.22) and ‘memory, attention and decision processes’ (mean=2.73, SD=0, 95% CI 2.73, 2.73). The only facilitator (where the mean score exceeded 5.0) was ‘beliefs about consequences’ (mean=5.74, SD=0.12, 95% CI 5.50, 5.98). A summary of the TDF findings is shown in table 3. Barriers and facilitators to fit note completion are illustrated in figure 1.

**Table 3:** Responses for items within Theoretical Domains Framework (TDF) domains

TDF domain	Statements	Number of question responses(% total n=198)*	Meanquestion score (SD)	95% CI for question	Mean domain score (SD)	95% CI for domain
Knowledge	1. I am aware of the content of an effective fit note	112 (57%)	4.64 (2.06)	0.59, 8.69	4.36(0.68)	3.03, 5.68
	2. I am aware of the objectives of a fit note	113 (57%)	5.39 (1.51)	2.42, 8.36		
	3. I know what my responsibilities are, with regards to a fit note	109 (55%)	4.08 (2.15)	−0.13, 8.29		
	4. I know how to certify a fit note	110 (56%)	3.66 (2.26)	−0.76, 8.08		
	5. I know when to certify a fit note	109 (55%)	4.00 (2.24)	−0.39, 8.39		
Skills	1. I have received training regarding how to certify fit notes	108 (55%)	3.06 (2.37)	−1.59, 7.72	3.32(0.75)	1.84, 4.80
	2. I have received training regarding how to review fit notes	109 (55%)	2.83 (2.13)	−1.34, 7.01		
	3. I have the skills needed to certify fit notes	109 (55%)	4.44 (2.18)	0.16, 8.72		
	4. I have been able to practice certifying fit notes	109 (55%)	2.93 (2.31)	−1.60, 7.47		
Social/professional role and identity	1. Certifying fit notes is part of my role	105 (53%)	4.00 (2.27)	−0.45, 8.45	4.30(0.44)	3.44, 5.16
	2. It is my responsibility to certify fit notes using specific protocols/guidelines	102 (52%)	4.09 (2.04)	0.09, 8.09		
	3. Certifying fit notes is consistent with other aspects of my job	105 (53%)	4.80 (2.01)	0.87, 8.73		
Beliefs about capabilities	1. I am confident that I can certify fit notes for my patients using specific protocols/guidelines	101 (51%)	4.31 (1.87)	0.64, 7.97	4.12(0.24)	3.65, 4.58
	2. I am capable of certifying fit notes even when little time is available	98 (49%)	4.16 (2.00)	0.25, 8.08		
	3. I have the confidence to certify fit notes, even when other service providers I work with are not doing this	97 (49%)	4.38 (2.07)	0.32, 8.45		
	4. I have the confidence to certify fit notes even when my patients are not receptive	97 (49%)	4.11 (1.94)	0.31, 7.92		
	5. I have personal control over certifying fit notes	95 (48%)	4.02 (2.30)	−0.48, 8.52		
	6. For me, certifying a fit note is easy	95 (48%)	3.72 (1.99)	−0.18, 7.61		
Optimism	1. In uncertain times, when I certify fit notes I usually expect that things will work out okay	82 (41%)	3.79 (1.72)	0.42, 7.16	3.92(0.15)	3.64, 4.21
	2. When I certify fit notes, I feel optimistic about my job in the future	76 (38%)	4.08 (1.67)	0.80, 7.35		
	3. I do not expect anything will prevent me from certifying fit notes	91 (46%)	3.89 (1.76)	0.44, 7.34		
Beliefs about consequences	1. I believe certifying fit notes will lead to benefits for my patients	105 (53%)	5.66 (1.47)	2.78, 8.53	5.74(0.12)	5.50, 5.98
	2. I believe certifying fit notes will benefit public health (i.e., health of the whole population)	104 (53%)	5.62 (1.47)	2.73, 8.50		
	3. In my view, certifying fit notes is useful	105 (53%)	5.88 (1.35)	3.23, 8.54		
	4. In my view, certifying fit notes is worthwhile	105 (53%)	5.81 (1.33)	3.20, 8.42		
Reinforcement	1. I get recognition from management at the organisation where I work, when I certify fit notes	69 (35%)	3.35 (3.81)	0.09, 6.60	3.81(0.76)	2.32, 5.29
	2. When I certify fit notes, I get recognition from my colleagues	64 (32%)	3.39 (1.54)	0.37, 6.41		
	3. When I certify fit notes, I get recognition from those who it impacts	69 (35%)	4.68 (1.55)	1.65, 7.72		
Intentions	1. I intend to certify fit notes for each/every one of my patients	78 (39%)	4.47 (1.93)	0.69, 8.26	4.38(0.15)	4.09, 4.66
	2. I will definitely certify fit notes for each/every one of my patients	76 (38%)	4.21 (1.77)	0.74, 7.68		
	3. I have a strong intention to certify fit notes for each/every one of my patients	76 (38%)	4.45 (1.75)	1.01, 7.89		
Goals	1. Compared to my other tasks, certifying fit notes is a higher priority on my agenda	89 (45%)	2.89 (1.56)	−0.16, 5.94	3.22(0.51)	2.21, 4.22
	2. Compared to my other tasks, certifying fit notes is an urgent item on my agenda	88 (44%)	2.95 (1.71)	−0.39, 6.30		
	3. I have clear goals related to certifying fit notes for each of my patients	82 (41%)	3.80 (2.00)	−0.11, 7.72		
Memory, attention and decision processes	1. Certifying fit notes is something I do automatically	80 (40%)	2.73 (1.90)	−0.99, 6.44	2.73(0)	
Emotion	1. I am able to certify fit notes without feeling anxious	80 (40%)	4.20 (1.94)	0.40, 8.00	4.47(0.25)	3.98, 4.96
	2. I am able to certify fit notes without feeling distressed or upset	78 (39%)	4.69 (1.90)	0.96, 8.42		
	3. I am able to certify fit notes, even when I feel stressed	78 (39%)	4.53 (1.90)	0.81, 8.25		
Environmental context and resources	1. In the organisation I work, all necessary resources are available to allow me to certify fit notes	91 (46%)	3.37 (2.22)	−0.99, 7.73	3.57(0.22)	3.14, 4.01
	2. I have support from the management of the organisation to certify fit notes	84 (42%)	3.65 (2.13)	−0.51, 7.82		
	3. The management of the organisation I work for are willing to listen to any problems I have relating to certifying fit notes	82 (41%)	3.89 (2.01)	−0.04, 7.82		
	4. The organisation I work for provides the opportunity for training to certify fit notes	91 (46%)	3.35 (2.10)	−0.77, 7.48		
	5. The organisation I work for provides sufficient time for me to certify fit notes	74 (37%)	3.59 (1.90)	−0.13, 7.32		
Social influences	1. People who are important to me think that I should certify fit notes	84 (42%)	4.14 (1.69)	0.84, 7.45	4.50(0.26)	3.99, 5.01
	2. People whose opinion I value would approve of me certifying fit notes	92 (46%)	4.66 (1.75)	1.23, 8.09		
	3. I can count on support from colleagues whom I work with when things get tough when certifying fit notes	79 (40%)	4.72 (1.68)	1.43, 8.01		
	4. Colleagues whom I work with are willing to listen to my problems with regards to certifying fit notes	77 (39%)	4.47 (1.74)	1.06, 7.87		
Behavioural regulation	1. I have a detailed plan of how I will certify fit notes	77 (39%)	3.65 (2.01)	−0.29, 7.59	3.96(0.70)	2.60, 5.33
	2. I have a detailed plan of how to certify fit notes when patients who are in hospital/attend the service are not receptive	78 (39%)	3.38 (1.75)	−0.05, 6.82		
	3. I have a detailed plan of how I will certify fit notes when there is little time	77 (39%)	3.43 (1.85)	−0.20, 7.06		
	4. It is possible to adapt how I certify fit notes to meet my needs as a healthcare provider	78 (39%)	4.36 (1.85)	0.73, 7.99		
	5. Certifying fit notes is compatible with other aspects of my job	96 (48%)	5.00 (1.90)	1.28, 8.72		

Boxes shaded in red=barriers; green=facilitator.

* Not all participants completed the TDF specific questions. Percentages have been rounded to the nearest whole number*.*

#### Barriers

##### Skills

Scores were low relating to training in certifying fit notes (mean=3.06, SD=2.37, 95% CI −1.59, 7.72), reviewing fit notes (mean=2.83, SD=2.13, 95% CI −1.34, 7.01) and having been able to practice certifying fit notes (mean=2.93, SD=2.31, 95% CI −1.34, 7.01). There was, however, a more positive response to the statement ‘I have the skills needed to certify fit notes’ (mean=4.44, SD=2.18, 95% CI 0.16, 8.72).

##### Goals

The ‘goals’ domain is related to the importance afforded to fit note certification. The lowest scoring statements were as follows: ‘compared to my other tasks, certifying fit notes is a higher priority on my agenda’ (mean=2.89, SD=1.56, 95% CI −0.16, 5.94) and ‘certifying fit notes is an urgent item on my agenda’ (mean=2.95. SD=1.71, 95% CI −0.39, 6.30). The highest score was in response to the statement, ‘I have clear goals related to certifying fit notes for each of my patients’ (mean=3.80, SD=2.00, 95% CI −0.11, 7.72).

##### Memory, attention and decision processes

This domain had the lowest score. It was based on responses to just one statement that ‘certifying fit notes is something I do automatically’. It had a mean score of 2.73 (SD=1.90, 95% CI −0.99, 6.44) indicating that certifying fit notes was not part of most participants’ day-to-day work.

### Facilitator

#### Beliefs about consequences

This was the highest-scoring domain and the only one that scored sufficiently highly to be deemed a facilitator. Findings indicate that participants believe that certifying fit notes would lead to benefits for patients (mean=5.66, SD=1.47, 95% CI 2.78, 8.53) and public health (mean=5.62, SD 1.47, 95% CI 2.73, 8.50). Statements such as ‘certifying fit notes is useful’ (mean=5.88, SD=1.35, 95% CI 3.23, 8.54) and ‘certifying fit notes is worthwhile’ (mean=5.81, SD=1.33, 95% CI 3.20, 8.42) also received high scores.

### TDF scores according to professional role

The TDF domains ‘skills’ and ‘memory, attention and decision processes’ were identified as barriers across all the healthcare professionals. See table 3 for a detailed summary of scores.

In the domain ‘goals’, OTs had a mean score of 3.70 (SD=0.40, 95% CI 2.91, 4.49) and, therefore, would not be classed as a barrier (i.e., score of 3.5 or less) for that group individually. However, pharmacists scored particularly low (mean=2.22, SD=0.30, 95% CI 1.62, 2.82), which reduced the mean domain score to 3.22 (SD=0.51, 95% CI 2.21, 4.22). This indicates a lack of goals relating to fit notes and that for most participants, particularly pharmacists, certifying fit notes was perceived as neither a priority nor urgent.

Similarly, pharmacists’ scores met the criteria for barriers (i.e., below 3.5) for more domains than other professional groups. This included ‘social/professional role and identity’ (mean=3.09, SD=0.44, 95% CI 2.22, 3.95), ‘beliefs about capabilities’ (mean=3.46, SD=0.38, 95% CI 2.72, 4.20), ‘environmental context and resources’ (mean=3.10, SD=0.32, 95% CI 2.48, 3.72) ‘behavioural regulation’ (mean=3.33, SD=0.22, 95% CI 2.90, 3.77) and ‘intentions’ (mean=2.77, SD=0.12, 95% CI 2.54, 2.99). In each of these domains, the scores from the other professional groups were largely consistent (all exceeding 3.5), although insufficient to be considered a facilitator (i.e., not 5.0 or above). This may be a reflection of the low percentage of pharmacists in our study with fit note experience (n=4, 10%).

There was just one domain which scored above 5.0 for all healthcare professions surveyed, indicating that it is a potential facilitator for certifying fit notes. This domain was ‘beliefs about consequences’ which had a mean domain score of 5.74 (SD=0.12, 95% CI 5.50, 5.98) in which OTs scored particularly highly (mean=6.21, SD=0.13, 95% CI 5.96, 6.46). This shows that most participants felt that certifying fit notes was both useful and worthwhile and would lead to benefits for patients and public health (i.e., health of the whole population). Table 4 illustrates the difference in TDF scores across professional groups.

**Table 4:** TDF domains by professional groups

TDF domains	Nurses(n=20)		Occupational therapists (n=34)		Pharmacists(n=19)		Physiotherapists (n=40)	
	Mean score(SD)	95% CI	Mean score(SD)	95% CI	Mean score(SD)	95% CI	Mean score(SD)	95% CI
Knowledge	4.36(0.46)	3.47, 5.25	4.53(0.75)	3.07, 5.99	3.85(0.85)	2.18, 5.52	4.47 (0.64)	3.22, 5.73
Skills	3.43(0.63)	2.19, 4.66	3.40(0.85)	1.74, 5.06	2.81(0.92)	1.03, 4.58	3.48(0.66)	2.18, 4.78
Social/professional role and identity	4.71(0.45)	3.82, 5.59	4.24(0.35)	3.56, 4.92	3.09(0.44)	2.22, 3.95	4.84(0.51)	3.85, 5.84
Beliefs about capabilities	4.26(0.44)	3.41, 5.12	4.28(0.19)	3.09, 4.66	3.46(0.38)	2.72, 4.20	4.19(0.30)	3.61, 4.78
Optimism	3.79(0.06)	3.66, 3.91	4.18(0.32)	3.56, 4.80	3.87(0.30)	3.29, 4.45	3.85(0.46)	2.96, 4.74
Goals	3.08(0.69)	1.72, 4.43	3.70(0.40)	2.91, 4.49	2.22(0.30)	1.62, 2.82	3.46(0.61)	2.27, 4.64
Memory, attention and decision processes	3.13(0)	–	3.22(0)	–	1.50(0)	–	2.69(0)	–
Environmental context and resources	3.91(0.31)	3.30, 4.53	3.59(0.42)	2.77, 4.42	3.10(0.32)	2.48, 3.72	3.63(0.22)	3.21, 4.06,
Social influences	4.55(0.31)	3.95, 5.15	4.76(0.37)	4.03, 5.50	4.12(0.37)	3.40, 4.84	4.37(0.27)	3.84, 4.90
Emotion	4.60(0.58)	3.47, 5.73	4.40(0.16)	4.07, 4.72	4.13(0.23)	3.68, 4.59	4.60(0.20)	4.21, 5.00
Behavioural regulation	4.05(0.60)	2.86, 5.23	4.02(0.91)	2.24, 5.81	3.33(0.22)	2.90, 3.77	4.15(0.74)	2.70, 5.61
Beliefs about consequences	5.46(0.22)	5.03, 5.89	6.21(0.13)	5.96, 6.46	5.09(0.25)	4.60, 5.59	5.71(0.11)	5.49, 5.92
Reinforcement	3.87(0.76)	2.38, 5.37	4.05(0.70)	2.68, 5.43	3.82(0.81)	2.23, 5.41	3.54(0.73)	2.11, 4.98
Intentions	4.58(0.25)	4.09, 5.08	4.48(0.16)	4.16, 4.81	2.77(0.12)	2.54, 2.99	4.86(0.16)	4.55, 5.16

Boxes shaded in red=barriers; green=facilitator.

TDF, theoretical domains framework.

A comparison between group A (respondents who completed at least one TDF-specific question, n=114) and group B (those who did not complete any TDF-specific questions, n=83) is shown in online supplemental file 2. Comparison between the groups reveals that more participants in the group that completed the TDF-specific questions (group A) had received training to certify fit notes (n=48, 44%) compared with the group that did not complete the TDF questions (group B; n=16, 22%). More group A participants also had experience of completing fit notes (n=36, 32%), whereas group B had lower completion rates (n=10, 12%). It is possible that those without training in, or experience of fit note completion may have felt they lacked the knowledge or skills to complete the TDF-specific questions. It is also possible that the TDF-specific questions may not have seemed salient to this group, despite at least some TDF-specific questions not requiring experience of completing fit notes.

### Qualitative data

Participants provided a total of 95 free-text comments; 56 were in response to being asked about barriers affecting their ability to certify fit notes and 39 were about facilitators for fit note certification and suggestions for improvement. The findings relating to both barriers and facilitators are shown in table 5, with reference to the relevant TDF domains where applicable.

**Table 5:** Theoretical Domains Framework (TDF) (adapted from Atkins *et al*^24^) with illustrative examples from study participants

TDF domain	Component constructs	Examples from data
1. Knowledge	Procedural knowledge; knowledge of task environment.	*“I wasn’t even aware I was legally allowed to do [fit notes].’ (Pharmacist, NHS/Private).‘Not commonly known that nurses can now certify fit notes.” (Nurse, NHS).“Patients still tend to ask doctors.” (Physiotherapist, NHS).“Little knowledge or experience in the emergency department about completing [fit notes]“ (Nurse, NHS).*
2. Skills	Competence; ability; interpersonal skills; practice.	*“No training within my trust for non-medics to certify fit notes.” (Nurse, NHS)“I would not be willing to take this on as an additional duty without training.” (Pharmacist, NHS/Self-employed)“I know the patients well to certify the fit note.” (Physiotherapist, NHS)“Training and support from OT management and practice manager has been crucial.” (OT, NHS)*
3. Social/professional role and identity	Social identity; professional boundaries; group identity; leadership; organisational commitment	*“Don’t think it really fits with my role.” (Pharmacist, NHS)“We have been told by management that fit notes aren’t our responsibility.” (Physiotherapist, NHS)“I do not certify fit notes, the medics(…)do this.” (Physiotherapist, NHS)*
4. Beliefs about capabilities	Self-confidence; perceived competence; self-efficacy; self-esteem; empowerment; professional confidence.	*“I deliver vocational rehab so it’s an integral part of my role.” (OT, NHS/Self-employed)“OTs have perfect skills to make the note meaningful looking at functional activity suggesting modifications for work. Personally, I would see this as an asset to my role.” (OT, NHS)*
5. Optimism	Optimism; pessimism; identity	*“If it were to become part of my role, I would feel confident in doing so, with the guidance and support of my organisation.” (OT)“Not had the opportunity to do training yet. It will be very useful in my post.” (Physiotherapist, NHS).*
6. Beliefs about consequences	Outcome expectancies; anticipated regret; consequences.	*“Not sure how it would fit with a time pressured role - our speciality is so diluted at times, taking on further 'holistic tasks' I feel could risk the specialism of care we provide”. (Physiotherapist, NHS).*
7. Reinforcement	Rewards; (not) valued; incentives; punishment; consequences; contingencies.	[Things could be improved by] “*Allocating time and promotion and being valued for doing it within organisation”. (Physiotherapist, NHS)*
8. Intentions	Stability of intentions; stages of change model.	*“My employer does not recognise or promote this as part of advanced/consultant level practice, sadly.” (Nurse, NHS)“I know we could do it but as yet there has been no policy or guidance on how AHPs can do this.” (OT, NHS/private)*
9. Goals	Priorities; target setting; action planning; implementation intention.	*“In my clinical area (acute neuro) it would not be a priority.” (Physiotherapist, NHS)“I would be very happy to certify fit notes after training but this whole agenda has not been even raised or prioritised since the law changed.” (OT, NHS)*
10. Memory, attention and decision-making processes	Ability to retain information, focus selectively on aspects of the environment and choose between alternatives.	Not reported.
11. Environmental context and resources	Environmental stressors; material resources; organisational culture/climate; salient events/critical incidents; barriers/facilitators.	*“IT issues in terms of remote access and not able to download electronic Med 3.” (OT, NHS)“No additional time is provided.” (Nurse, NHS)“If it were to become a big part of my role [and] take away from the day-to-day work, then would need more manpower.” (OT, NHS).“As an organisation we do not yet have any procedures or protocols to follow.” (OT, NHS)*
12. Social influences	Social pressures; social norms; group conformity; social comparisons; group norms; social support; power; intergroup conflict; alienation; group identity; modelling.	*“We have a programme where we do the online e-learning and sit with a competent fit note issuer to ensure confidence is built”. (Nurse, NHS)*
13. Emotion	Fear; anxiety; stress; depression; positive/negative affect.	Not reported.
14. Behavioural regulation	Self-monitoring; breaking habits; action-planning.	Not reported.

The main themes arising from analysing the qualitative data are presented below.

#### Knowledge and awareness of legislation change

Several participants claimed to have little, or no, knowledge about the change in legislation to allow NOPPs to certify fit notes. This was often attributed to poor communication within their organisation. In addition, participants highlighted a lack of awareness among patients who they believed would still ask the GP for their fit note. This lack of knowledge among staff and patients indicated major barriers to certifying fit notes.

#### Training

The domain ‘skills’ was found to be a barrier in the quantitative data with particularly low scores relating to training and practising certifying fit notes. In the free-text comments, training was the most frequently mentioned word (53 mentions). Being unable to access training was seen as a barrier, whereas completing fit note training was considered a facilitator, particularly because participants suggested that their confidence would be improved by undergoing appropriate training.

Suggestions for how training might be improved included having support and encouragement from managers and allocated time. Participants also suggested having more in-house training, opportunities for case-based discussions (face to face or through Teams) and involving colleagues with fit note experience. These suggestions indicate the importance of peer support which falls within the TDF domain, ‘social influences’.

#### Role

There were several free-text comments relating to the TDF domain ‘social/professional role and identity’. Barriers relating to this theme included believing, or being told that, it was not a responsibility of their role to certify fit notes.

Despite this, most of the free-text comments relating to the TDF domains ‘social/professional role’ and ‘beliefs about capabilities’ were positive, although not scoring sufficiently high enough to be considered as facilitators for fit note completion.

The comments suggested that participants largely welcomed the opportunity to certify fit notes and did not consider it to be outside of the current requirements of their role. Many felt that they had already performed the skills needed to complete a fit note, and some referred to prior experience of completing the AHP report as a facilitator. For example:

‘As a Mental Health [OT], we have always explored vocational rehab as a part of our role. The fit note is just an extension of [the AHP fitness for work report]. Therefore, it did not feel like a large step.’ (OT, NHS).

#### Environmental context and resources

This domain scored very low in the quantitative data, indicating that a lack of resources was a barrier to certifying fit notes. Free-text comments provided examples including IT issues (being unable to access electronic versions of fit notes), insufficient time and staff shortages. However, concerns about lack of time and insufficient staff levels may stem from wanting to provide an effective service for their patients, as this comment indicates:

‘Not sure how it would fit with a time pressured role—our speciality is so diluted at times, taking on further 'holistic tasks' I feel could risk the specialism of care we provide.’ (Physiotherapist, NHS)

Free-text responses provided further insights into another barrier which was having no opportunity to certify fit notes. This is sometimes related to the participant’s place of work. For example, most participants worked in hospitals, but there were higher numbers of participants in general practice who had undergone training and had experience of certifying fit notes (as shown in table 2). Possible reasons for this discrepancy were suggested in free-text comments indicating that medics were still expected to certify fit notes in secondary care. For example:

‘Currently an ACP (advanced clinical practitioner) in inpatient mental health. No training within my trust for non-medics to certify fit notes. Still very much being left to medics to complete despite the change.’ (Nurse, NHS)

Another factor was the patient population that NOPPs were working with. For example, fit notes may not be considered a priority where most patients are retired or students.

Also, in the TDF domain of ‘environmental context’, there were frequent mentions of a lack of guidance, policies and procedures. This suggests that barriers at organisational level are preventing some NOPPs certifying fit notes.

In contrast, the issue of organisational/managerial support was raised by participants in response to being asked about potential and actual facilitators. Examples included having agreed policies, guidance and training, along with supportive teams and management.

More commonly, participants indicated the need for changes at organisation/management level to enable NOPPs to certify fit notes, such as raising awareness, managerial approval and providing protocols to follow. The following was a typical comment in response to the survey question asking what could be improved:

‘National push with managers being more aware of it [training], then providing adequate time for completing it.’ (Physiotherapist, NHS).

This comment encapsulates the environmental barriers faced by many participants.

## Discussion

This study set out to explore the experiences and knowledge of NOPPs around certifying fit notes. However, the main finding was that over 12 months since the introduction of legislation allowing NOPPs to certify fit notes, only a quarter of our survey participants had done so. This is the first study to our knowledge to incorporate feedback from all of the professions included in the new legislation around fit note certification, and therefore, this small-scale exploratory study represents an important contribution to the literature in this area.

Most participants worked in hospitals, but fewer had experience of fit note completion compared with participants working in primary care who had most fit note experience. This finding contrasts with previous studies of task reallocation from doctors to nurses which found that hospitals were more supportive of expanding nursing practice.^21^

The three main barriers to fit note completion reported by our survey respondents were in the TDF domains ‘skills’, ‘goals’ and ‘memory, attention and decision processes’. Closer scrutiny of the results indicates that the low scores related to all these TDF domains stem from an absence of guidelines and policies to indicate which healthcare professionals should do the training, where they should access it, and what support would be provided to NOPPs undertaking these additional duties. In contrast to previous studies which uncovered barriers^20^ to the adoption of clinical guidelines,^23 29^ findings from the current study suggest that clinical guidance has yet to be developed in response to legislation change allowing NOPPs to certify fit notes. This indicates that further research should focus on organisation-level barriers to providing guidelines in respect of fit note legislation. In this study, OTs and physiotherapists were most likely to have accessed training possibly because online courses were promoted by their professional bodies. In other respects, ‘skills’ were highly scored, indicating that many respondents felt that they possessed the necessary skills needed to certify fit notes. This raises the question of why participants felt they possessed skills but still believed they needed training. It may be that they were referring to workability assessments and providing RTW advice (as suggested in the free-text comments) or, similar to the first contact practitioner physiotherapists in Black *et al*’s study,^20^ there were concerns about the legal aspects of fit note completion.

The TDF category of ‘memory, attention and decision process’ which was reported as a barrier, was based on one question where respondents disagreed with the statement that ‘certifying fit notes is something I do automatically’. This was answered by just 40% of respondents and is probably related to the similarly low number of respondents who had completed fit note training. Also, in the TDF domain ‘goals’, findings indicated a low level of priority or urgency afforded to certifying fit notes by NOPP respondents, mirroring responses in a study of GPs.^13^ This suggests that there is a need to raise awareness among healthcare professionals around the role of fit notes in enabling a ‘safe, smooth and rapid’ RTW, and the associated benefits of doing so.^30^

Other barriers highlighted by respondents related to environmental factors and resources, including technology (such as being unable to access electronic versions of the form) and shortage of time. Similarly, two systematic reviews identified time allocated for training and in medical consultations as the most frequently mentioned barrier to implementing clinical practice guidelines.^21 23^ Conversely, access to technology and time for training are the main facilitators for implementing clinical practice guidelines.^23 29^ Therefore, these findings support recommendations that adequate resources be made available to facilitate fit note training and completion.^18^

The one facilitator identified in the TDF analysis was in the domain ‘beliefs about consequences’ which showed a positive perception of the value of fit notes for patient care and public health. As healthcare professionals’ positive attitudes towards changes to clinical practice guidelines have been recognised as an important enabler,^23^ the likelihood of NOPPs adopting fit note legislation in future may be enhanced.

This exploratory study had several key strengths. It is the first to explore the experiences and opinions of all professionals newly able to certify fit notes, and though limited by a small sample, most of whom had no fit note experience, it provides important evidence to inform and support future training, guidelines and resource allocation. Despite the low response rate, we achieved our required sample size, including a diverse sample of healthcare providers working in different settings across the UK, which allowed us to capture a range of perspectives on the factors influencing fit note certification. Our findings present both quantitative and qualitative data from the survey, providing useful insights into the barriers and facilitators to fit note completion and the context for their implementation. These issues were explored in more depth in subsequent interviews with the same research population (reported separately). A further strength is that data collection and analysis were guided by the TDF framework, which adds theoretical rigour to the study.

However, our study did have some limitations. Although using an online survey promoted through social media enabled access to a wide range of NOPPs employed in a variety of settings, it meant that a response rate could not be calculated because we do not know how many health professionals were reached by the survey. We were also unable to collect any data on non-responders, so we do not know how they differed from responders, and it is possible that those with experience of fit note completion and those with more positive views towards it were more likely to complete the survey than the general population of NOPPs. If this was the case, our estimate of the frequency of fit note completion may be an overestimate, while barriers to fit note completion may be underestimated and facilitators overestimated compared with those in the general population of NOPPs. Therefore, implementation of fit note completion by NOPPs may be even less successful than our findings suggest.

Furthermore, the original intention when designing the survey was to explore NOPPs’ experiences of completing fit notes. When there was a low response rate, we altered the criteria to include NOPPs who had no experience of completing fit notes and/or had not undergone any fit note training. Although this resulted in a low completion rate of TDF-related questions, it allowed us to gauge the level of awareness of the change in legislation allowing NOPPs to complete fit notes. Moreover, free-text comments suggested areas for further exploration in a subsequent interview study with the same research population.

Considering the lack of awareness of the changes to fit note legislation, we feel that the response was satisfactory in that we achieved our proposed sample size. However, we acknowledge that we can only make cautious conclusions given the small sample compared with the number of NOPPs employed in the UK. We, therefore, present this research as an exploratory study which can be built on in subsequent research.

The study is also limited by low numbers of question responses. The unanticipated lack of fit note experience among the sample meant that responses to the TDF-specific questions varied from 32% to 57%. Questions relating to the TDF domains ‘reinforcement’, ‘intentions’ and ‘emotions’ had the fewest responses, probably because the questions required some level of fit note experience. Future studies should include separate questions for those with, and without, fit note experience.

Pharmacists were difficult to recruit and were the least likely profession to have attended fit note training. This may reflect the limited opportunities for pharmacists to issue fit notes, possibly due to their lack of access to patients’ medical records which may prevent them from safely providing a fit note.^17^ Finally, although free-text comments allowed participants to expand on their responses to survey questions, as with all such surveys, it was difficult to contextualise their comments. However, this limitation has been addressed through subsequent interviews allowing further exploration of facilitators and barriers to fit note completion in particular contexts. Results of the interview study will be reported separately.

## Conclusions

This exploratory study, which included all professional groups newly able to certify fit notes, indicates that legislation allowing NOPPs to undertake fit note certification has not been widely implemented. Most barriers appear to be at management/organisation level with poor communication about the legislation change and how it should be implemented. The main facilitator was participants believing that certifying fit notes was appropriate for their professional role and that they possessed the necessary skills to offer RTW advice. Findings suggest that organisational-level changes are necessary to improve communication, develop guidelines and provide the necessary resources to enable NOPPs to fulfil this important task. This will help to ensure that the potential advantages of enabling NOPPs to certify fit notes are realised, maximising benefits at individual patient and HCP levels. It could also potentially benefit patient care and healthcare systems more generally by relieving pressures on GPs and improving availability of GP appointments. Supporting patients to RTW and providing advice to patients and their employers will potentially yield financial benefits for individuals as well as having wider economic advantages for society.

## Supplementary material

10.1136/bmjopen-2024-092211 online supplemental file 1

10.1136/bmjopen-2024-092211 online supplemental file 2

## Data Availability

Data are available on reasonable request.
