# Supplementary material for: Experiences and knowledge of nurses, occupational therapists, pharmacists and physiotherapists about certifying fit notes: a UK-wide survey
Source: BMJ Open. 2025 May 15;15(5):e092211. doi: 10.1136/bmjopen-2024-092211 (PMC12083426; doi:10.1136/bmjopen-2024-092211)
Supplement: online supplemental file 1 [file bmjopen-15-5-s001.pdf]

Supplementary File 1: Online survey

Participant Information

Experiences and knowledge of pharmacists, nurses, occupational therapists and physiotherapists certifying fit notes.

Chief Investigator: Dr Jade Kettlewell, Centre for Academic Primary Care, Applied Health Research Building, University of Nottingham, Faculty of Medicine & Health Sciences

Research Ethics Ref: FMHS 230-0323

This study aims to explore the barriers and facilitators to the certification of fit notes and experiences of healthcare professionals in certifying fit notes.

You are invited to take part because you are:
• Employed or self-employed as a pharmacist, occupational therapist, physiotherapist or nurse
• Hold a current registration with a relevant professional healthcare regulator

Please read through the participant information sheet via the link below before agreeing to participate. Taking part is entirely voluntary.

[link to participant information sheet]

If you decide to continue with the survey, then please click the 'I consent to take part' button at the bottom of this page. By ticking this box, you are consenting to take part in this survey. Your responses will remain anonymous and any information you share will be treated confidentially.

If you have any questions about the study or your participation in this survey, please contact Dr Jade Kettlewell, jade.kettlewell2@nottingham.ac.uk

○ I consent to take part

Demographic Information

1. What is your profession?

- ○ Occupational therapist
- ○ Physiotherapist
- ○ Nurse
- ○ Pharmacist

---

2. Do you have experience of certifying fit notes?

- ○ No
- ○ Yes

3. How many fit notes have you certified to date? Please provide an estimate if not sure.

	0 30 60 90 120 150 180 210 240 270 300	Not applicable
Number of fit notes certified	______________________	☐

4. Did you receive any training to certify fit notes? If yes, please provide details.

- ○ No
- ○ Yes ______________________________

5. Do you work within the NHS, private practice or are you self-employed? Please select all that apply.

- ◯ NHS
- ◯ Private practice
- ◯ Self employed

6. Which setting(s) do you currently work in? Please select all that apply.

- ◯ General practice
- ◯ Community team
- ◯ Hospital
- ◯ Community pharmacy
- ◯ Health centre
- ◯ Patients' homes
- ◯ Other (please specify) ______________________________

7. What is your main area of expertise?

- ◯ Musculoskeletal
- ◯ Mental health
- ◯ Neurological conditions
- ◯ Cancer
- ◯ Injuries
- ◯ Pain
- ◯ Cardiovascular

- ○ No specialist area – general patient health
- ○ Other (please specify) ____

8. How many years have you been qualified?

- ○ 0-5 years
- ○ 6-10 years
- ○ 11-15 years
- ○ Over 15 years

9. We would like to understand more about how you currently feel about certifying and reviewing fit notes. There are no right or wrong answers. Please select the response that relates most closely to your current situation and experience.

	Strongly disagree (1)	Disagree (2)	Somewhat disagree (3)	Neither agree nor disagree (4)	Somewhat agree (5)	Agree (6)	Strongly agree (7)	Not applicable
I am aware of the content of an effective fit note	○	○	○	○	○	○	○	○
I am aware of the objectives of a fit note	○	○	○	○	○	○	○	○
I know what my responsibilities are, with regard to certifying a fit note	○	○	○	○	○	○	○	○
I know how to certify a fit note	○	○	○	○	○	○	○	○
I know when to certify a fit note	○	○	○	○	○	○	○	○
I have received training regarding how to certify fit notes	○	○	○	○	○	○	○	○
I have received training regarding how to review fit notes	○	○	○	○	○	○	○	○
I have the skills needed to certify fit notes	○	○	○	○	○	○	○	○
I have been able to practice certifying fit notes	○	○	○	○	○	○	○	○
Certifying fit notes is part of my role	○	○	○	○	○	○	○	○
It is my responsibility to certify fit notes using specific protocols/guidelines	○	○	○	○	○	○	○	○
Certifying fit notes is consistent with other aspects of my job	○	○	○	○	○	○	○	○

10. We would like to understand more about your confidence of certifying or reviewing fit notes. There are no right or wrong answers. Please select the response that relates most closely to your current situation and experience.

	Strongly disagree (1)	Disagree (2)	Somewhat disagree (3)	Neither agree nor disagree (4)	Somewhat agree (5)	Agree (6)	Strongly agree (7)	Not applicable
I am confident that I can certify fit notes for my patients using specific protocols/guidelines	○	○	○	○	○	○	○	○
I am capable of certifying fit notes even when little time is available	○	○	○	○	○	○	○	○
I have the confidence to certify fit notes, even when other professionals I work with are not doing this	○	○	○	○	○	○	○	○
I have the confidence to certify fit notes even when my patients are not receptive	○	○	○	○	○	○	○	○
I have personal control over certifying fit notes	○	○	○	○	○	○	○	○
For me, certifying fit notes is easy	○	○	○	○	○	○	○	○
In uncertain times, when I certify fit notes I usually expect that things will work out okay	○	○	○	○	○	○	○	○
When I certify fit notes, I feel optimistic about my job in the future	○	○	○	○	○	○	○	○
I do not expect anything will prevent me from certifying a fit note	○	○	○	○	○	○	○	○

11. We would like to understand more about how you currently feel about certifying fit notes. There are no right or wrong answers. Please select the response that relates most closely to your current situation and experience.

	Strongly disagree (1)	Disagree (2)	Somewhat disagree (3)	Neither agree nor disagree (4)	Somewhat agree (5)	Agree (6)	Strongly agree (7)	Not applicable
Compared to my other tasks, certifying fit notes is a higher priority on my agenda	○	○	○	○	○	○	○	○
Compared to my other tasks, certifying fit notes is an urgent item on my agenda	○	○	○	○	○	○	○	○
I have clear goals related to certifying fit notes for each of my patients	○	○	○	○	○	○	○	○
Certifying fit notes is something I do automatically	○	○	○	○	○	○	○	○
In the organisation I work, all necessary resources are available to allow me to certify fit notes	○	○	○	○	○	○	○	○
I have support from the management of my organisation to certify fit notes	○	○	○	○	○	○	○	○
The management of the organisation I work for are willing to listen to any problems I have relating to certifying fit notes	○	○	○	○	○	○	○	○
The organisation I work for provides the opportunity for training to certify fit notes	○	○	○	○	○	○	○	○
The organisation I work for provides sufficient time for me to certify fit notes	○	○	○	○	○	○	○	○
People who are important to me think that I should certify fit notes	○	○	○	○	○	○	○	○
People whose opinion I value would approve of me certifying fit notes	○	○	○	○	○	○	○	○
I can count on support from colleagues whom I work with when things get tough when certifying fit notes	○	○	○	○	○	○	○	○
Colleagues whom I work with are willing to listen to my problems with regards to certifying fit notes	○	○	○	○	○	○	○	○

	Strongly disagree (1)	Disagree (2)	Somewhat disagree (3)	Neither agree nor disagree (4)	Somewhat agree (5)	Agree (6)	Strongly agree (7)	Not applicable
I am able to certify fit notes without feeling anxious	◯	◯	◯	◯	◯	◯	◯	◯
I am able to certify fit notes without feeling distressed or upset	◯	◯	◯	◯	◯	◯	◯	◯
I am able to certify fit notes, even when I feel stressed	◯	◯	◯	◯	◯	◯	◯	◯
I have a detailed plan of how I will certify a fit note	◯	◯	◯	◯	◯	◯	◯	◯
I have a detailed plan of how to certify fit notes when patients are not receptive	◯	◯	◯	◯	◯	◯	◯	◯
I have a detailed plan of how I will certify fit notes when there is little time	◯	◯	◯	◯	◯	◯	◯	◯
It is possible to adapt how I certify fit notes to meet my needs as a healthcare provider	◯	◯	◯	◯	◯	◯	◯	◯
Certifying fit notes is compatible with other aspects of my job	◯	◯	◯	◯	◯	◯	◯	◯

12. We would like to understand more about your beliefs and motivations for certifying fit notes. There are no right or wrong answers. Please select the response that relates most closely to your current situation and experience.

	Strongly disagree (1)	Disagree (2)	Somewhat disagree (3)	Neither agree nor disagree (4)	Somewhat agree (5)	Agree (6)	Strongly agree (7)	Not applicable
I believe certifying fit notes will lead to benefits for my patients	○	○	○	○	○	○	○	○
I believe certifying fit notes will benefit public health (ie. health of the whole population)	○	○	○	○	○	○	○	○
In my view, certifying fit notes is useful	○	○	○	○	○	○	○	○
In my view, certifying fit notes is worthwhile	○	○	○	○	○	○	○	○
I get recognition from management at the organisation where I work when I certify fit notes	○	○	○	○	○	○	○	○
When I certify fit notes, I get recognition from my colleagues	○	○	○	○	○	○	○	○
When I certify fit notes, I get recognition from those who it impacts	○	○	○	○	○	○	○	○
I intend to certify a fit note for every patient that requires one	○	○	○	○	○	○	○	○
I will definitely certify a fit note for every patients that requires one	○	○	○	○	○	○	○	○
I have a strong intention to certify a fit note for every patient that requires one	○	○	○	○	○	○	○	○

13. We would like to know more about your experiences of certifying fit notes. Please could you tell us about any barriers or issues that affect your ability to certify fit notes? Provide as much detail as possible, your responses will remain anonymous.

14. Is there anything that could facilitate (or has facilitated) your certification of fit notes? (e.g. allocated time to complete them, training, support). Please tell us how you think things could be improved.

Prize Entry

Optional
To thank you for taking the time to complete this survey, you have the option to enter the prize draw to win a £50 Amazon gift voucher.

If you would like to be entered into the prize draw, please enter your email address in the box below. Your email address will not be associated with the survey responses and will remain anonymous.
You will be contacted by a researcher if you win the prize.

If you do not wish to enter, please proceed.

Optional
As part of this research, we want to interview up to 20 healthcare professionals like you to understand more about your experiences and opinions of certifying fit notes. We want to know what impact the new task of certifying fit notes has on your role, the extent to which any fit note training has met your needs and the impact of widening the range of professionals completing fit notes has on the usefulness of the fit note.

If you are interested in taking part in an interview please leave your contact details below. A member of the research team will be in touch to provide further information.
