# Supplementary material for: Experiences and knowledge of nurses, occupational therapists, pharmacists and physiotherapists about certifying fit notes: a UK-wide survey
Source: BMJ Open. 2025 May 15;15(5):e092211. doi: 10.1136/bmjopen-2024-092211 (PMC12083426; doi:10.1136/bmjopen-2024-092211)
Supplement: online supplemental file 2 [file bmjopen-15-5-s002.docx]

**Supplementary File 2**

Comparison of Group A (participants that completed at least one TDF-specific question) and Group B (participants that did not complete any TDF-specific questions).

Percentages rounded to nearest whole number.

| **Profession** | **Group A Total** | **Group B Total** | **Experience of Completing Fit Notes** | | **No Experience of Completing Fit Notes** | | **Received Training to Certify Fit Notes** | | **No Training to Certify Fit Notes** | |
| --- | --- | --- | --- | --- | --- | --- | --- | --- | --- | --- |
|  |  |  | **Group A**  **(n=114)** | **Group B**  **(n=83)** | **Group A**  **(n=114)** | **Group B**  **(n=83)** | **Group A**  **(n=108)*** | **Group B**  **(n=74)**** | **Group A**  **(n=108)*** | **Group B**  **(n=74)**** |
| **Nurse** | 20 | 24 | 7  (35%) | 5  (21%) | 13  (65%) | 19  (79%) | 9  (47%) | 5  (24%) | 10  (53%) | 16  (76%) |
| **Occupational Therapist** | 34 | 15 | 11  (32%) | 1  (7%) | 23  (68%) | 14  (93%) | 16  (48%) | 6  (46%) | 17  (52%) | 7  (54%) |
| **Pharmacist** | 19 | 20 | 3  (16%) | 1  (5%) | 16  (84%) | 19  (95%) | 4  (24%) | 2  (11%) | 13  (76%) | 17  (89%) |
| **Physiotherapist** | 41 | 24 | 15  (37%) | 3  (13%) | 26  (63%) | 21  (88%) | 19  (49%) | 3  (14%) | 19  (49%) | 18  (86%) |
| **All professions** | 114^a^ | 83 | 36  (32%) | 10  (12%) | 78  (68%) | 73  (88%) | 48  (44%) | 16  (22%) | 59  (55%) | 58  (78%) |

^a^  1 participant did not report profession

* Missing data for 6 participants for this question: nurse (n=1), occupational therapist (n=1), pharmacist (n=2), physiotherapist (n=2)

** Missing data for 9 participants for this question: nurse (n=3), occupational therapist (n=2), pharmacist (n=1), physiotherapist (n=3)
