# Supplementary material for: Experiences and knowledge of nurses, occupational therapists, pharmacists and physiotherapists about certifying fit notes: a UK-wide survey
Source: BMJ Open. 2025 May 15;15(5):e092211. doi: 10.1136/bmjopen-2024-092211 (PMC12083426; doi:10.1136/bmjopen-2024-092211)
Supplement: Supplementary file 3 [file bmjopen-2024-092211.reviewer_comments.pdf]

PEER REVIEW HISTORY

BMJ Open publishes all reviews undertaken for accepted manuscripts. Reviewers are asked to complete a checklist review form and are provided with free text boxes to elaborate on their assessment. These free text comments are reproduced below.

ARTICLE DETAILS

Title (Provisional)

Experiences and knowledge of nurses, occupational therapists, pharmacists, and physiotherapists about certifying fit notes: A UK-wide survey

Authors

Kettlewell, Jade; Trusson, Diane; Powers, Katie; Drummond, Avril; Anderson, Claire; Phillips, Gill; Holmes, Jain; Radford, Kate; Pahl, Nick; Martin, Shan; Timmons, Stephen; Kendrick, Denise

---

VERSION 1 - REVIEW

**Reviewer**	**1**
**Name**	**MADAN, IRA**
**Affiliation**	**GUYS AND ST THOMAS NHS FOUNDATION TRUST**
**Date**	**10-Sep-2024**
**COI**	**Nil**

The study seeks to establish the behavioural determinants that influence nurses, pharmacists, physios and OTs prescribing fit notes. The sampling frame is very broad and includes social media. The authors acknowledge that the denominator is unknown.

Unfortunately, I do not consider the study to be sufficiently scientifically robust to publish. The main problems are:

1. A small sample size given the numbers of nurses/ physios/ pharmacists/ OTs in England/UK the response rate must have been tiny. I disagree with the authors' comment in the discussion that a strength of the study is the large number of participants.

2. Given the tiny response rate, the study is subject to large and unquantifiable selection bias.

3.No detail is given on how the sample size for the study was calculated.

4. The authors fail to state the degree of uncertainty around the results (no confidence intervals given).

5. The authors used TDF questions to ascertain the answers to their research question , but these questions were not applicable in 76% of the participants.

Given the above biases and flaws in the study the results are unreliable. I therefore cannot recommend for publication. It would have been better if the authors had used robust qualitative methods to answer their study question.

Reviewer 2

Name Bornhöft, Lena

Affiliation Department of Health and Rehabilitation, Institute of Neuroscience and Physiology, Sahlgrenska Academy at University of Gothenburg, Gothenburg, Sweden

Date 21-Oct-2024

COI I have no competing interests.

I would like to thank the authors for a very clear and well-written paper describing the effects of legal changes to which professions may issue fit notes in the UK.

The only revision I would like to point out as necessary is in Table 4. Here the red squares indicating barriers have been marked incorrectly. Instead of colouring squares for pharmacists mean + SD, you have coloured OT-SD and pharmacist-mean. Also for Goals - Nurse-SD should be red and not OT-SD.

I also wonder if there is an explanation for why so many without experience of issuing fit notes participated when the introduction to the survey states clearly that you were looking for professionals with experience of issuing fit notes. Perhaps the effects of this can be discussed. Or perhaps a comparison of results for those with experience of issuing fit notes as compared to those without experience would be of interest.

VERSION 1 - AUTHOR RESPONSE

**Reviewer 1.**<br>1. Unfortunately, I do not consider the study to be sufficiently scientifically robust to publish. The main problems are:<br><br>A small sample size given the numbers of nurses/ physios/ pharmacists/ OTs in England/UK the response rate must have been tiny. I disagree with the authors' comment in the discussion that a strength of the study is the large number of participants.	Thank you to the reviewer for this comment. We agree that the number of participants is not large in relation to the number of nurses/ physios/ pharmacists/ OTs in England. As we were unable to directly approach health professionals through professional bodies, academic and professional networks and social media we do not know how many health professionals were reached by the survey and therefore, as with any survey conducted using these methods, it was not possible to calculate a response rate. We have changed the text in the discussion to read:<br><br>*'This exploratory study had several key strengths. It is the first to explore the experiences and opinions of all professionals newly able to certify fit notes, and though limited by a small sample, most of whom had no fit note experience, it provides important evidence to inform and support future training, guidelines and resource allocation. Despite the low response rate, we achieved our required sample size, including a diverse sample of healthcare providers working in different settings across the UK, which allowed us to capture a range of perspectives on the factors influencing fit note certification.'*<br>*'Considering the lack of awareness of the changes to fit note legislation, we feel that the response was satisfactory in that we achieved our proposed sample sized. However, we acknowledge that we can only make cautious conclusions given the small sample compared to the number of NOPPs employed in the UK. We therefore present this research as an exploratory study which can be built on in subsequent research.'*
2. Given the tiny response rate, the study is subject to large and unquantifiable selection bias.	We thank the reviewer for this comment. As explained in point 1 above, we are not able to calculate a response rate. This was acknowledged as a limitation in the discussion, but we have strengthened this as below. In addition, we agree with the reviewer that, as with any survey, bias can arise because those who participate in the survey may differ from those who do not and estimating the likely extent and impact of this bias is difficult because we do not know the response rate or characteristics of non-responders. We have changed the text in the discussion to better acknowledge these limitations.<br><br>*'Although using an online survey promoted through social media enabled access to a wide range of NOPPs employed in a variety of settings, it meant that a response rate could not be calculated because we do not know how many health professionals were reached by the survey. We were also unable to collect any data on non- responders, so we do not know how they differed from responders, and it is possible that those with experience of fit note completion and those with more positive views towards it were more likely to complete the survey than the general population of NOPPs. If this was the case our estimate of the frequency of fit note completion may be an overestimate, whilst barriers to fit note completion may be underestimated and facilitators overestimated compared to those in the general population of NOPPs.*<br>*Therefore, implementation of fit note completion by NOPPs may be even less successful than our findings suggest.'*

3. No detail is given on how the sample size for the study was calculated.	We thank the reviewer for this comment. The paper did include the following text:<br>*‘A sample size calculation indicated that a sample of 19 per professional group (i.e. 76) was needed to estimate an overall domain mean for each group with 95% confidence and a precision of 0.5, based on a standard deviation (SD) of 1.’*<br>We agree that more detail is needed and have amended the text below and added references, so the text now reads:<br>*‘A sample size calculation indicated that a sample of 19 per professional group (i.e. 76) is required to estimate an overall TDF domain mean with 95% confidence and a precision of 0.5, based on a standard deviation (SD) of 1[26]. The SD is based on a previous study using a TDF survey to identify behavioural factors affecting implementation[27]. TDF mean domain scores can range from 1 to 7. With 19 participants per group and a SD of 1, the study would have 80% power at the 5% significance level to detect a 1-point difference in mean domain scores between two professional groups[26].’*
4. The authors fail to state the degree of uncertainty around the results (no confidence intervals given).	Thank you for this comment. We have calculated the 95% confidence intervals for each question and each domain as requested. This has been added to Table 3 and reported in text where appropriate.
5. The authors used TDF questions to ascertain the answers to their research question, but these questions were not applicable in 76% of the participants.	On reflection, we realise that our statement regarding participants who did not have experience of completing fit notes was misleading because despite the lack of fit note experience among participants, there were no questions that were only answered by 24% of the sample.<br><br>We have now inserted the following wording in the text: *‘The unanticipated lack fit note experience amongst the sample meant that responses to the TDF-specific questions varied from 32% to 57%. Questions relating to the TDF domains ‘reinforcement’, ‘intentions’ and ‘emotions’ had the fewest responses, probably because the questions required some level of fit note experience. Future studies should include separate questions for those with, and without, fit note experience.’*<br><br>We have also compared the participants that completed the TDF specific questions and those that didn’t to explore possible explanations for why questions were not completed. We have added a comparison table as a supplementary file and also added the following to the results section:<br>*‘The proportion of responders completing TDF-specific questions ranged from 32% to 57%.’*<br>*‘A comparison between Group A (respondents who completed at least one TDF-specific question, n=114) and Group B (those who did not complete any TDF-specific questions, n=83) is shown in Supplementary file 2.*<br>*Comparison between the groups reveals that more participants in the group that completed the TDF-specific questions (Group A) had received training to certify fit notes (n=48, 44%) compared to the group that didn’t complete the TDF questions (Group B; n=16, 22%). More group A participants also had experience of completing fit notes (n=36, 32%), whereas Group B had lower completion rates (n=10, 12%). It is possible that those without training in, or experience of fit note completion may have felt they lacked the knowledge or skills to complete the TDF-specific questions. It is also possible that the TDF-specific questions may not have seemed salient to this group, despite at least some TDF-specific questions not requiring experience of completing fit notes.’*

6. Given the above biases and flaws in the study the results are unreliable. I therefore cannot recommend for publication. It would have been better if the authors had used robust qualitative methods to answer their study question.	Although we acknowledge the reviewer's concerns, we have been encouraged by the editor to respond to the feedback and resubmit an amended manuscript. We believe that despite its limitations, the survey indicates that fit note legislation has not been widely implemented. This alone is an important finding, given the potential benefits of extending fit note completion to NOPPs as set out in the paper. The survey being reported here was the first stage of our research; we followed up with an interview study which allowed us to explore reasons for the low level of engagement with the new legislation. A paper reporting the findings from the qualitative study will be published separately.
Reviewer 2<br>1. I would like to thank the authors for a very clear and well-written paper describing the effects of legal changes to which professions may issue fit notes in the UK.	Thank you for these encouraging comments.
2. The only revision I would like to point out as necessary is in Table 4. Here the red squares indicating barriers have been marked incorrectly. Instead of colouring squares for pharmacists mean + SD, you have coloured OT-SD and pharmacist-mean. Also for Goals - Nurse-SD should be red and not OT-SD.	Thank you for pointing out these errors. We have now corrected them in the revised document.
3. I also wonder if there is an explanation for why so many without experience of issuing fit notes participated when the introduction to the survey states clearly that you were looking for professionals with experience of issuing fit notes. Perhaps the effects of this can be discussed. Or perhaps a comparison of results for those with experience of issuing fit notes as compared to those without experience would be of interest.	Thank you for alerting us to this error. Unfortunately, the copy of the survey which was included with the manuscript was not the most recent one. The revised version of the survey did not require participants to have experience of completing fit notes. This version will be submitted with the other revised documents.<br>Nevertheless, the following text has been added to the discussion to address this issue.<br>'Furthermore, the original intention when designing the survey was to explore NOPPs' experiences of completing fit notes. When there was a low response rate, we altered the criteria to include NOPPs who had no experience of completing fit notes and/or had not undergone any fit note training. Although this resulted in a low completing rate of TDF-related questions, it allowed us to gauge the level of awareness of the change in legislation allowing NOPPs to complete fit notes. Moreover, free-text comments suggested areas for further exploration in a subsequent interview study with the same research population.'<br><br>We have also included a comparison of results (shown in supplementary file 1) between those that did and did not complete the TDF specific questions and added the following to the results section. This highlights that a number of participants did not complete all questions as the majority did not have any fit note experience nor had they received any training:<br>'A comparison between Group A (respondents who completed at least one TDF-specific question, n=114) and Group B (those who did not complete any TDF-specific questions, n=83) is shown in Supplementary file 2.

	Comparison between the groups reveals that more participants in the group that completed the TDF-specific questions (Group A) had received training to certify fit notes (n=48, 44%) compared to the group that didn't complete the TDF questions (Group B; n=16, 22%). More group A participants also had experience of completing fit notes (n=36, 32%), whereas Group B had lower completion rates (n=10, 12%). It is possible that those without training in, or experience of fit note completion *may have felt they lacked the knowledge or skills to complete the TDF-specific questions. It is also possible that the TDF-specific questions may not have seemed salient to this group, despite at least some TDF-specific questions not requiring experience of completing fit notes.'*

VERSION 2 - REVIEW

Reviewer 2

Name Bornhöft, Lena

Affiliation Department of Health and Rehabilitation, Institute of Neuroscience and Physiology, Sahlgrenska Academy at University of Gothenburg, Gothenburg, Sweden

Date 06-Jan-2025

COI

The authors have responded adequately to my concerns. As the aim of the study was to identify rather than quantify behavioural determinants that influence NOPPs' certification of fit notes, it seems to me that the relatively low number of responders is not a major problem. I chose minor revision as I was unable to find Figure 1.
